# Treatment of Pneumonia

**Published:** 1888-01

**Authors:** C. B. Leitner

**Affiliations:** Flora, Ala.


### TREATMENT OF PNEUMONIA.*

BY C. B. LEITNER, M. D., FLORA, ALA.

Having been requested to give my treatment of pneumonia, I do so with pleasure, believing it to be the most reliable course to pursue in this fearful disease. I base this belief upon the report of the statistics published of the mortality from this disease particularly in the middle States; knowing that physicians do not report a greater mortality than really takes place in their practice.

In the first place, it is of the utmost importance that the disease should be properly diagnosed, which cannot be done without the use of the stethoscope and pleximeter—the hand can be used for the latter. Why should we be so particular about the diagnosis? Because the bronchial tubes way may be inflamed, with bloody expectoration, as it is in pneumonia, but not dull over the substance of the lungs, which can be ascertained by percussion. If this inflammation only were present, it would be pneumonia in

*Read before the Bullock County (Ala.) Medical Society.

a few days by continuity of structure. Here again the stethoscope comes into requisition. Satisfied that every intelligent physician can distinguish pneumonia from bronchitis, though it may be in the finer tubes of the bronchia, I will proceed with my treatment.

I take one-half ounce of ground Rad Senega, this being the very best stimulating expectorant, ten or twelve beans of Jaborandi, the best diaphoretic, add to this twenty-four ounces of water, simmer, not boil, down to sixteen ounces of water. If it is below the measurement, add hot water until it amounts to sixteen ounces when strained. Add to this two drachms of nitrate of potash, which is a good diuretic, and sixty (60) grains of quinine. Keep in an earthen pitcher or bowl. Dose, one tablespoonful every two hours until diaphoresis takes place. Be governed by the effects of the remedy in producing sweating.

In the first place, the patient's bowels should be moved freely with a mercurial purgative, such as calomel, rhubarb, ipecacuanha, with counter irritation over the diseased part, by cupping and the use of the blister. If the patient is plethoric, let the cupping be very free. If the urine be scanty, I draw a large cup of buchu tea, giving a tablespoonful in combination with the expectorant or at intervals until that trouble is corrected. I use gum opium at night to produce sleep. There is another point which should be well watched, as it is deceptive; that is, acute pain in the thoracic pleura, as it may be remote from the inflammation of the lungs, and it flies from point to point if it be not permanent pleuro-pneumonia. Of course the blistering and cupping must include the point of pain on the thorax. I cannot, as you will observe, give directions as to how often the tea should be given, as the indications of the skin will be the monitor governing the intervals of giving and the quantity. When I find the skin cool and pleasant I have been accustomed to giving ammoniated julep, but good whisky or brandy has answered and will answer every purpose. Remember to be controlled by the use of the stethoscope. I use no arterial sedative unless the fever is very high and the skin very dry, then I use nothing but aconite; no veratrum.

The blister should be kept alive by applying a poultice of slippery elm and wheat-bran constantly. For convenience have two poultices; when one is on, the other can be laid for several hours in a little warm water, when the one can take the place of the other. Remember to give your patient rest at night with opium pills. I cannot too highly recommend this treatment, as it has seldom or never failed in my hands, notwithstanding the formidable character of the disease.

There will be some complications in this disease, which the good sense of the physicians must combat.

I will be pleased to hear from any one of you through correspondence, if any point in this article is not fully understood.

